# Biological Impact of the TSHβ Splice Variant in Health and Disease

**DOI:** 10.3389/fimmu.2014.00155

**Published:** 2014-04-07

**Authors:** John R. Klein

**Affiliations:** ^1^Department of Diagnostic and Biomedical Sciences, University of Texas Health Science Center at Houston, Houston, TX, USA

**Keywords:** alternatively spliced, bone marrow, hormone, immune–endocrine, isoform, pituitary, thyroid, thyrotropin

## Abstract

Thyroid stimulating hormone (TSH), a glycoprotein hormone composed of α and β chains, is produced by thyrotrope cells of the anterior pituitary. Within the conventional endocrine loop, pituitary-derived TSH binds to receptors in the thyroid, resulting in the release of the thyroid hormones thyroxine (T_4_) and triiodothyronine (T_3_). T_4_ and T_3_ in turn regulate nearly every aspect of mammalian physiology, including basal metabolism, growth and development, and mood and cognition. Although TSHβ has been known for years to be produced by cells of the immune system, the significance of that has remained largely unclear. Recently, a splice variant of TSHβ (TSHβv), which consists of a truncated but biologically functional portion of the native form of TSHβ, was shown to be produced by bone marrow cells and peripheral blood leukocytes, particularly cells of the myeloid/monocyte lineage. In contrast, full-length native TSHβ is minimally produced by cells of the immune system. The present article will describe the discovery of the TSHβv and will discuss its potential role in immunity and autoimmunity, inflammation, and bone remodeling.

## TSH and the Immune System

The hypothalamus–pituitary–thyroid (HPT) axis is an integrated hormone network that is essential for maintaining mammalian physiology, basal metabolism, growth, development, mood, and cognition. Thyroid stimulating hormone (TSH) belongs to a set of glycoprotein hormones that includes lutropin, follitropin, and chorionic gonadotropin. All four hormones consist of an α-subunit and a non-covalently bound β-subunit (1). Hormone specificities are dictated by the β-subunit. Thyrotropin-releasing hormone (TRH) is produced in the hypothalamus and transported to the anterior pituitary via the superior hypophyseal artery, where it induces the release of TSH. TSH travels via the circulation to the thyroid, binds to the TSH receptor (TSHR) on thyroid follicular cells, and induces the secretion of the thyroid hormones, thyroxine (T_4_) and triiodothyronine (T_3_). Although T_4_ is the predominant thyroid hormone present in the circulation, it is principally a pro-hormone of more biologically active T_3_, which is generated following conversion of T_4_ to T_3_ in the tissues by deiodinases. Extensive feedback mechanisms, in particular the levels of circulating TSH, T_4_ and T_3_, control TRH and TSH output.

The mouse TSHβ gene consists of five exons. The human TSHβ gene consists of three exons. The coding regions are located in exons 4 and 5, and exons 2 and 3, in mouse and human TSHβ, respectively. There is considerable homology at both the gene and protein levels between human and mouse TSHβ (2). In both species, TSHβ consists of 138 amino acids, 118 of which comprise the native TSHβ protein with a 20 amino acid signal peptide. Evidence that TSH is produced by cells of the immune system dates back over three decades (3–5). Since then, TSH has been shown to have extensive involvement in immune regulation, development, and effector function activity in primary and secondary lymphoid cell populations, as well as in mucosal sites in the intestine. A number of reviews have covered these topics (6–8). Additionally, an osteoprotective role for TSH has recently been reported in bone formation involving osteoblast generation and osteoclast destruction (9). The focus of the present review will be on the characterization and function of a recently described TSHβ splice variant (TSHβv) (10).

## Identification and Characterization of the TSHβv

Although TSH can be produced by both myeloid and lymphoid cells, myeloid cells in the bone marrow (BM) and peripheral leukocytes generated from those cells are the primary source of immune system TSH (6, 11–13). Intracellular staining for TSHβ and quantification of TSH synthesis by enzyme-linked assays revealed a CD11b^+^ cell population to be the predominant BM TSHβ-producing cell (14).

An early clue that immune system TSH may have a functional role in regulating metabolism came from *in vivo* studies in which mice expressing a transgenic T cell receptor for hen–egg lysozyme had transient suppression of circulating T_3_ and T_4_, and that there was an influx of CD11c^+^ cells into the thyroid following antigen exposure (15). Moreover, hypophysectomized (HPX) mice challenged with alloantigen had a significant increase in serum T_4_ levels (15). Because HPX mice are unable to make pituitary-derived TSH, the signal responsible for elevated levels of T_4_ appeared to have been derived from an extrapituitary source.

Trafficking studies in which BM cells from enhanced green fluorescent protein transgenic mice were used to reconstitute lethally irradiated syngeneic host animals demonstrated the presence of intrathyroidal leukocytes consisting of CD11b^+^ cells that did not express CD3, CD4, CD8α, CD19, CD40, Ly-6G, or F4/80, although a small proportion were CD11c^+^ (14). Trafficking to the thyroid occurred as early as 1 week post-BM reconstitution and continued until at least 20 weeks post-reconstitution (14). Direct evidence that intrathyroidal CD11b^+^ cells produced TSH was established by two-color staining of fresh-frozen thyroid tissue sections using anti-CD11b and anti-TSHβ antibodies (14).

**Figure 1:**
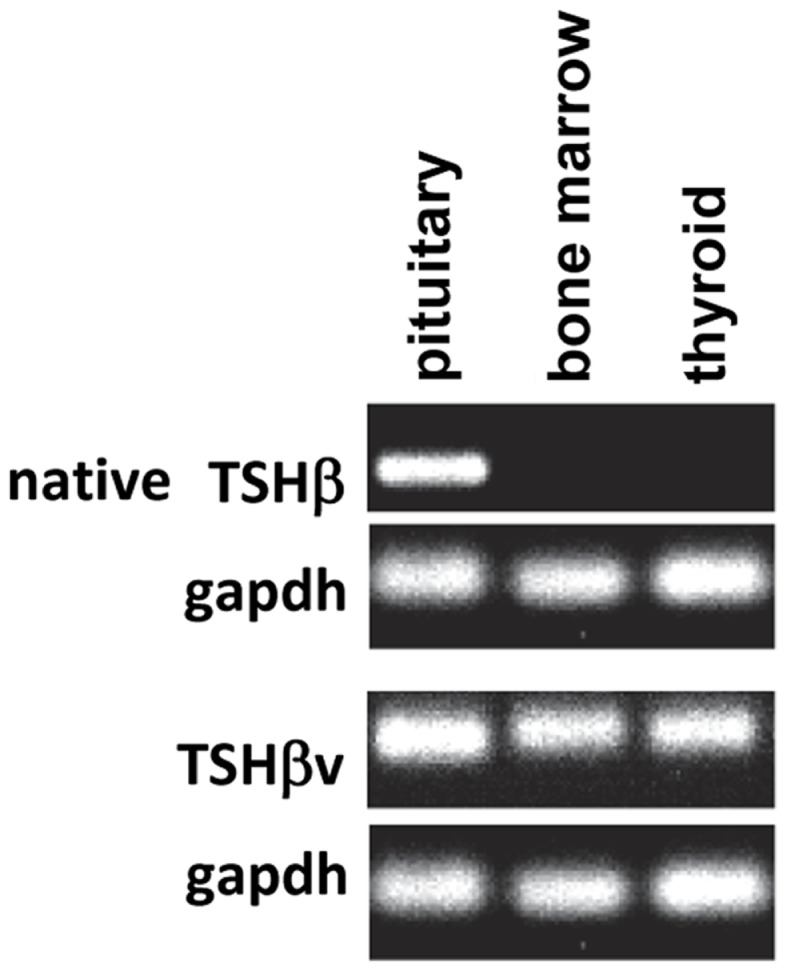
**PCR analysis of native TSHβ and TSHβv gene expression in mouse pituitary, bone marrow, and thyroid tissues**. Note the expression of the native form of TSHβ in the pituitary but not the bone marrow and thyroid, and the expression of the TSHβ in all three tissues. Presented data were derived from Ref. (10).

**Figure 2:**
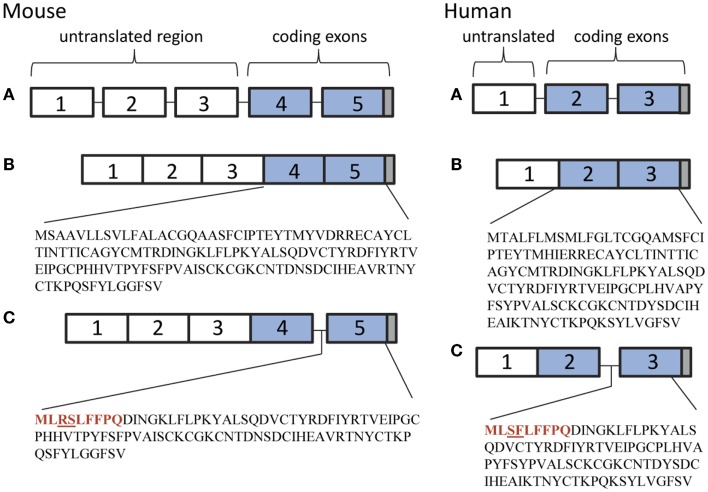
**(A)** Genetic organization of the mouse and human TSHβ gene. **(B)** The native TSHβ polypeptide in mice and humans is coded for by exons 4 and 5, and exons 2 and 3, respectively (blue boxes). **(C)** The TSHβv polypeptide is coded for by exon 5 in mice and exon 3 in humans. The 27 nucleotide 3′ end of intron 4 in mouse and intron 2 in human TSHβv codes for a nine amino acid signal peptide (shown in red) beginning with a methionine translational start site. Gray boxes represent stop codons.

While conducting a series of studies to assess the conditions under which TSH is produced in the thyroid, we observed that there was no amplification of the TSHβ gene in BM cells or thyroid tissues using primers targeted to the full-length mouse TSHβ transcript (all of exons 4 and 5) (10). Using a primer set that targeted the 3′ end of intron 4 and a downstream region just after the TAA stop codon of exon 5, conventional and qRT-PCR analyses were done from pituitary, thyroid, and BM tissues. A PCR product was detectable in the pituitary but not the BM or thyroid using primers for the full-length native transcript, whereas a PCR product was detected in the pituitary, the BM, and the thyroid using primers that targeted exon 5 (Figure 1). This suggested that alternative splicing of the TSHβ gene had occurred at or near the beginning of mouse exon 5, thus excluding exon 4 from the gene product. DNA sequencing of the PCR product revealed homology to exon 5 of the mouse TSHβ gene with a portion of intron 4 that was retained and contiguous with exon 5 (10). This consisted of 27 nucleotides from intron 4 beginning with an ATG start codon and was in-frame with exon 5 of mouse TSHβ. This coded for nine amino acids (MLRSLFFPQ) and a truncated protein comprising 71% of the native TSHβ molecule (10). Similar findings were obtained using human tissues (16). These are shown in Figure 2. However, the possibility also must be considered that transcription of TSHβv is due not to alternative splicing but to initiation of transcription from within introns 4 and 2 of mouse and human TSHβ, respectively.

**Figure 3:**
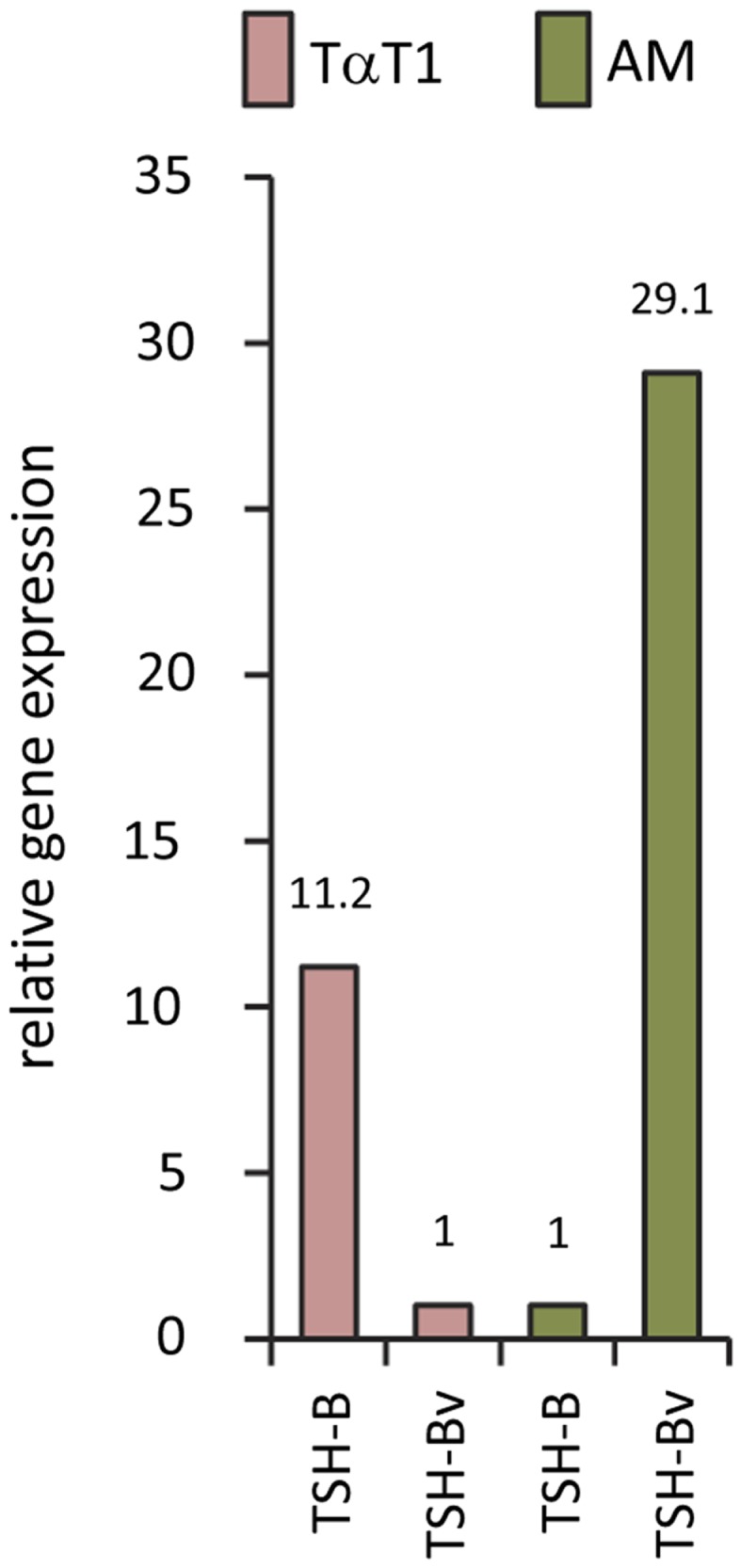
**Gene expression of native TSHβ and TSHβv in mouse TαT1 thyrotropic cells and the mouse AM macrophage cell line, showing low TSHβv gene expression in TαT1 cells and high expression in macrophages**.

Studies using the mouse TαT1 thyrotropic cell line and the mouse AM macrophage cell line demonstrated high levels of native TSHβ and minimal TSHβv in TαT1 cells, and low levels of native TSHβ and high levels of TSHβv in AM cells (Figure 3). Mouse BM-derived myeloid cells have been shown to preferentially express the TSHβv in CD11b^+^ M2 macrophages relative to M1 macrophages (17). Expression of the TSHβv is low in monocytes, neutrophils, and lymphocytes (17).

**Figure 4:**
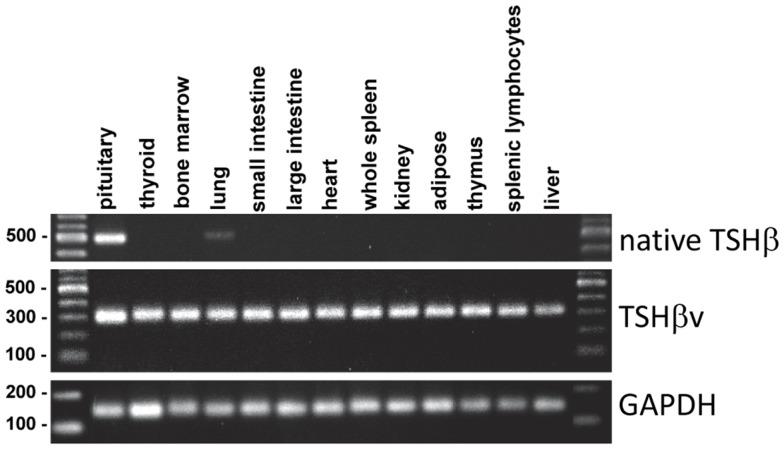
**Tissue distribution of native TSHβ and TSHβv in 13 mouse tissues**. Note the restricted expression of native TSHβ primarily in the pituitary, and the wide distribution of TSHβv throughout the other tissues.

In mice, the TSHβv transcript is present in tissues throughout the body, whereas the full-length native TSHβ transcript is largely restricted to the pituitary (Figure 4). The wide distribution of the TSHβv isoform likely does not reflect expression by the somatic tissues themselves, but may represent the presence of leukocytes, particularly CD11b^+^ cells within the circulation, that are embedded in those tissues, although this has yet to be formally demonstrated. The presence of trace amounts of native TSHβ gene expression in the lung is interesting but unclear at this time.

Evidence that the TSHβv protein is actively secreted comes from western blot studies using supernatants from CHO cells transfected with the mouse TSHβv gene (10), from western blots of serum from healthy persons (18), and from mass spectrometry analysis of peptides from BM cell culture supernatants (17). Co-immunoprecipitation experiments using recombinant human TSHα and TSHβv revealed dimerization of the TSHβv with TSHα (18), a condition that would be needed for optimal binding of the TSHβv to the TSHR.

## Role of the TSHβv in Health and Disease

### TSHβv during antigenic challenge and virus infection

That the TSHβv retains functional activity in terms of intracellular signaling has been established from *in vitro* studies of cAMP responses in mouse AM cells and rat FRTL thyroid follicular cells (10), and in Chinese hamster ovary cells transfected with the TSHR cultured in the presence of BM macrophages as a source of TSHβv (17).

**Figure 5:**
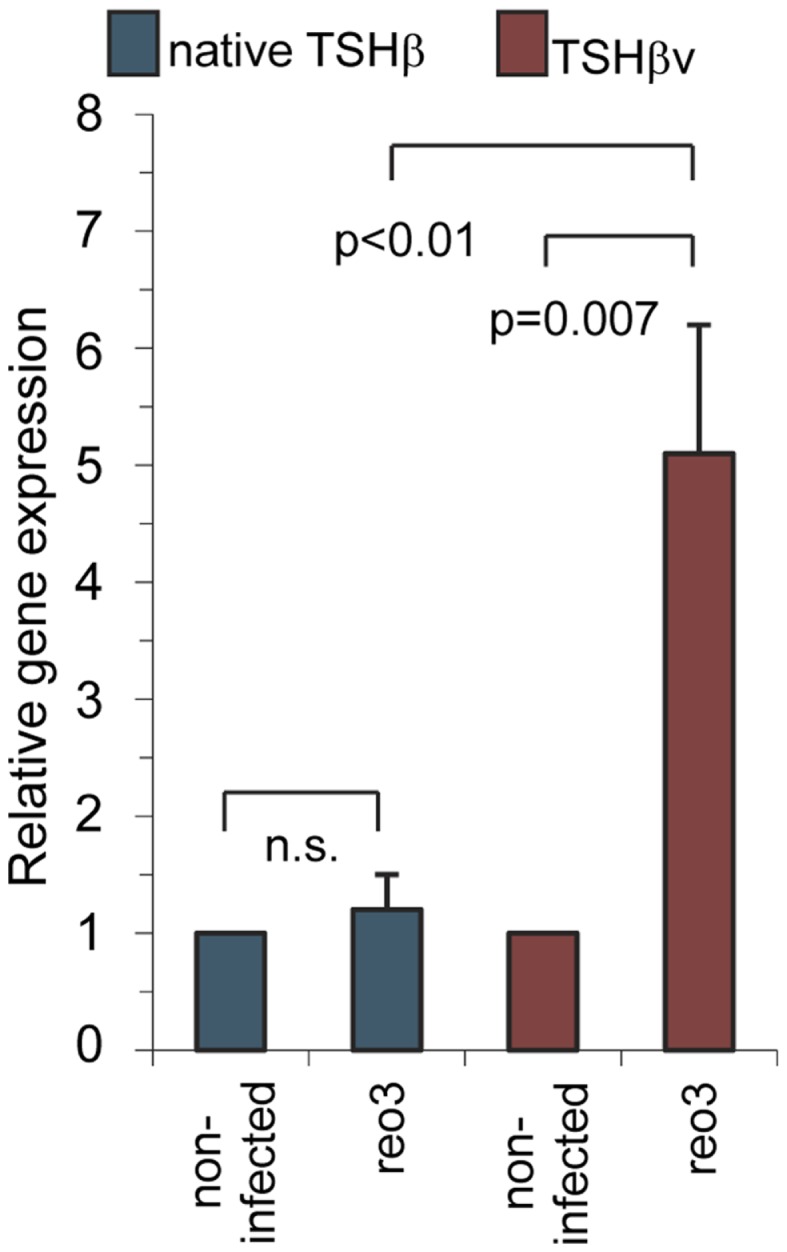
**Systemic reovirus infection induces gene expression of TSHβv but not native TSHβ in thyroid tissues**. Mice were infected intraperitoneally with 10^7.5^ plaque-forming units of reovirus serotype 3 Dearing strain. Tissues were assayed for gene expression 48 h post-infection. Presented data were derived from Ref. (10).

To determine if antigenic challenge, in this case virus infection, influences the expression levels of the TSHβv in the thyroid, C57BL/6 mice were infected intraperitoneally with serotype 3 reovirus. Thyroid tissues were isolated 48 h later. Virus infection had no effect on native TSHβ gene expression in the thyroid relative that of non-infected mice; however, there was a significant increase in TSHβv transcript levels in the thyroid of virus-infected mice (10) (Figure 5), indicating that the host response to infection was accompanied by a selective increase in intrathyroidal synthesis of the TSHβv. These findings, coupled with studies using alloantigen-primed mice (15), suggest that elevated levels of the TSHβv are produced in the thyroid during foreign antigen exposure. The effect of this may be to suppress circulating thyroid hormone production and lower the host metabolic activity during periods of infection by blocking native TSHβ binding. A model for this has been proposed (19). Interestingly, TSHβ synthesis also has been shown to be increased in the small intestine of mice following oral infection with reovirus (20) or rotavirus (21), although the form of TSHβ produced locally was not determined in those studies.

### TSHβv in chronic inflammation

Besides the involvement of immune system TSH during infection, there are a large number of human conditions with links to thyroid dysregulation that have yet to be fully understood, many of which have notable inflammatory components. These include Graves’ disease and Hashimoto’s thyroiditis (22), Graves’ ophthalmopathy (23, 24), Pendred’s syndrome (25), Lyme disease (26), inflammatory bowel disease (27), rheumatoid arthritis (28), systemic lupus erythematosus (29, 30), psoriasis (31), asthma (32), sepsis (33, 34), and hypothyroidism that may accompany type I interferon therapy (35–37).

Although much still needs to be done to establish a role for TSHβv in disease, some evidence for this already exists. In a study of patients with Hashimoto’s thyroiditis (HT), transcript levels of the TSHβv were higher in peripheral blood leukocytes (PBL) of HT patients compared to normal controls (18). Prednisone treatment of HT patients significantly reduced TSHβv transcript levels in patients having a short duration of disease (≤9 months) compared to patients with a long duration (≥18 months) or to controls. Consistent with that, TSHβv transcript levels in PBL of HT patients were reduced in a dose-dependent manner *in vitro* upon exposure to dexamethasone (18). These findings point to a potential involvement of the TSHβv in the pathogenesis of HT.

### TSHβv as a regulator of bone morphogenesis

Recent studies have identified an osteoprotective role for TSH involving osteoclast growth and osteoclast inhibition (9). Although early studies linking bone loss and thyroid function were largely regarded to be due to elevated thyroid hormone levels, studies using *Tshr*^−/−^ mice that were incapable of delivering a TSHR signal but were made hyperthyroid by T_4_ supplementation revealed a pattern of bone loss similar to that of hyperthyroid wild-type mice, thus implicating a failure of TSH signaling, not excessive thyroid hormone synthesis, as the cause of poor bone remodeling (38). Those findings now have been linked to the TSHβv as shown by the proximity of TSHβv-producing macrophages in mouse vertebral bone, by the capacity of macrophage-derived TSHβv to induce osteoblast formation, and suppression in the presence of anti-TSH antibody (17).

## Potential Clinical Involvement of the TSHβv in Health and Disease

The TSHβv – the first functional alternatively spliced form of TSHβ to be identified in mice and humans (10, 16, 39) – could have a multitude of here-to-fore unknown biological activities, which may be beneficial or detrimental to the host depending upon the clinical setting. Already, three potential candidates for this have been identified.

First, the TSHβv may contribute to the process by which thyroid hormone synthesis is regulated. Competitive binding of TSHβv to thyroid TSHR may block native TSHβ binding. Whether this occurs, or whether the TSHβv can preferentially displace native TSHβ or vice-verse, has yet to be demonstrated. Similarly, it will be of interest to determine the extent to which TSHβv and native TSHβ bind to discrete regions of the TSHR, and whether they differentially dimerize to the TSHα moiety. Competitive binding studies may help to elucidate this. Additionally, the fact that the TSHR is widely expressed in the BM and throughout the peripheral immune system (13, 21, 40–42), raises questions of whether those cells operate in some manner to regulate the amount of immune system-derived TSHβv that is available. Preliminary studies in our laboratory using recombinant mouse TSHβv suggest this leads to lower circulating T_4_ levels (Montufar-Solis and Klein, unpublished). Whether that effect is beneficial to the host remains to be determined; however, during acute infection, immune system-derived TSHβv may function as an alternative regulator of metabolism.

Second, continually dysregulated synthesis of TSHβv from cells of the immune system, possibly as a consequence of chronic inflammation due to the excessive accumulation of CD11b^+^ cells, could lead to HT. The TSHβv protein, which was shown to be present in sera of normal persons (18), may increase in chronic inflammatory conditions, resulting in a non-homeostatic tilt favoring the TSHβv isoform over native TSHβ. This was implied by the finding of increased gene expression levels of TSHβv in PBL of HT patients (18). Interestingly, hypothyroidism is an occasional complication of patients undergoing type I interferon therapy (35–37). Whether that reflects an imbalance between native TSHβ and TSHβv caused by an inflammatory response induced by interferon is unknown. Further studies will need to be done to address this.

Third, the beneficial effects of TSHβv produced by bone-associated M2 macrophages could be an on-going process throughout life linked to bone remodeling (17). This would provide a local source of TSH that could be modulated independently of pituitary TSH. Whether the numbers, or the production of the TSHβv, of BM-derived macrophages is changed during aging will be of interest to determine.

Clearly, a key feature of the TSHβv isoform is its immune system source. This provides a new and exciting insight into how two of the body’s major physiological systems, the immune system and the endocrine system, come together in a collaborative way in the maintenance of health, and in the potential for disease when disruption of that axis occurs.

## Conflict of Interest Statement

The author declares that the research was conducted in the absence of any commercial or financial relationships that could be construed as a potential conflict of interest.
